# Untapped Insight: A Longitudinal Qualitative Analysis of Older Adults’ Advice During the COVID-19 Pandemic

**DOI:** 10.1093/geroni/igac071

**Published:** 2022-11-16

**Authors:** Bryce Van Vleet, Heather R Fuller, Brittany Hofmann, Andrea Huseth-Zosel

**Affiliations:** Department of Human Development and Family Science, North Dakota State University, Fargo, North Dakota, USA; Department of Human Development and Family Science, North Dakota State University, Fargo, North Dakota, USA; Department of Human Development and Family Science & School of Business, North Dakota State University, Fargo, North Dakota, USA; Department of Public Health, North Dakota State University, Fargo, North Dakota, USA

**Keywords:** Coping, Generativity, Intergenerational communication, Life course, Pattern coding

## Abstract

**Background and Objectives:**

Due to a lifetime of experience, older adults are uniquely positioned to contribute advice and insight to others during a historical, societal crisis such as the coronavirus disease 2019 (COVID-19) pandemic. This qualitative study explores the solicited advice older adults offered their peers, family members, and communities throughout the first year of the pandemic.

**Research Design and Methods:**

A sample of 72 older adults aged 70–97 from Minnesota and North Dakota were asked what advice they would provide to others in June 2020 and again in April/May 2021. Participants were asked to provide advice on individual coping and how community members should support older adults during the pandemic, as well as how others should adjust after the pandemic. Responses were coded and developed into overarching themes.

**Results:**

Older adults advised others during the pandemic to *foster mental and physical well-being*, *develop positive life perspectives*, and *connect to others* as strategies to cope through the pandemic. Participants advised that after the pandemic people should *remain vigilant*, *return to normal*, and *emerge as better people*. Advice targeted to meeting the needs of older adults during the pandemic included: *adopt selfless attitudes*, *take intentional actions*, and *maintain balance*. A longitudinal approach revealed that advice remained consistent over time, despite the circumstances caused by COVID-19 changing.

**Discussion and Implications:**

Findings suggest that older adults utilize their life experiences and coping strategies as sources for drawing advice. These findings also suggest that older adults are sources of insight during crises. Future research should investigate additional advice older adults can offer and how willingly communities listen. Applied work should give older adults opportunities to provide far-reaching advice as well as develop interventions aimed at decreasing ageist perceptions of older adults in times of crisis.

**Translational Significance:** This study documented solicited advice of older adults during the coronavirus disease 2019 pandemic identifying themes of staying connected, being selfless, and exercising caution. These findings illuminated older adults as an untapped source of insight and guidance who have the potential to help foster resilience among younger community members during this pandemic or future societal crises. Additionally, findings of this study inform researchers and practitioners about the possible benefits of advice-giving in later life generally and during historical crises specifically, suggesting an area for innovative intervention given U.S. older adults are not often in a position to provide insight and advice widely.

## Background and Objectives

Older adults often draw from their lived experiences to provide advice and pass wisdom down to younger generations (Parisi et al., 2009). Additionally, in times of crisis older adults may lean on their lived experiences through historical time and events to cope (Aldwin & Igarashi, 2016; Lind et al., 2021; Settersten, 2017). The coronavirus disease 2019 (COVID-19) pandemic is one such crisis. This pandemic necessitated precautions including physical distancing for all ages (e.g., Teater et al., 2021). However, older adults faced greater risk of severe health consequences including mortality from COVID-19 and because of this, were disproportionally negatively affected by the pandemic (Harrison et al., 2021; van der Kaap-Deeder et al., 2021). Yet, older adults have displayed resiliency during the pandemic by utilizing life experience (Chan et al., 2022; Fuller & Huseth-Zosel, 2021) and adaptive coping strategies (Vannini et al., 2021) to cope with this crisis. This study sought to explore the advice older adults offer on how to cope with and emerge from this historical challenge.

### Advice and Wisdom of Older Adults

Older generations are often motivated to offer wisdom to younger generations as part of normative aging processes and intergenerational learning (Tabuchi & Miura, 2016). Wisdom is the knowledge, reasoning, and cognitive complexity accumulated over one’s life that is often applied to help others solve problems (Ardelt, 2011; Tabuchi & Miura, 2015). Wisdom is often developed through difficult life events such as health challenges and interruptions to social relationships (Igarashi et al., 2018), and can be shared with others through the dissemination of advice. Advice is “a recommendation on how to act, think, or feel in response to a problem” (MacGeorge, 2020, p. 287). In Western contexts, older adults have few opportunities outside of their family roles (i.e., parent, grandparent) to ­provide advice (Schafer & Upenieks, 2016; Spence et al., 2001).

When older adults have the opportunity to give advice, the process is linked with positive development such as contributing to life meaning and generativity (Schafer & Upenieks, 2016). Thus, advice provision can be beneficial for older adults. In adulthood, generally, receiving advice from others has been linked with the development of better relationships, social capital, better health, and higher-quality decision making (MacGeorge & Van Swol, 2018). These findings suggest that advice provision is indeed a mechanism to help others. Advice literature often focuses on everyday, unsolicited advice contexts such as in personal relationships (e.g., Branch & Hall, 2018; Hill, 2018) or workplaces and mentorships (e.g., Bonaccio & Paik, 2018; Zheng & North, 2020). There is also considerable focus in the advice literature from those living with chronic medical conditions (e.g., Kahana et al., 2009; Taylor et al., 1997) indicating that advice often takes place in the context of adversity. Taken together, these findings suggest that crises like the COVID-19 pandemic may provide an optimal setting to share wisdom through advice.

As both advice and wisdom are seen as helping younger generations, these constructs are most commonly linked to the concept of generativity (e.g., Parisi et al., 2009; Schafer & Upenieks, 2016; Tabuchi & Miura, 2015) in which an individual invests in the next generation (Erikson, 1950). Generativity can be expressed in several ways including through childrearing, mentoring, or participating in volunteer and/or political organizations (McAdams & de St. Aubin, 1992). While Erikson (1950) viewed generativity as occurring primarily in late middle adulthood, McAdams and de St. Aubin (1992) have asserted that generativity is not clearly tied to a specific point in adulthood; rather, it is an ongoing task of adulthood. Engagement in generativity is evident in older age (e.g., Parisi et al., 2009; Tabuchi & Miura, 2015). However, older adults are less likely to give advice as compared to midlife adults (Schafer & Upenieks, 2016), which suggests that advice provision may not be a common marker of older adulthood. Further, they are more likely than younger adults to value received advice (Bailey et al., 2021) which may be a result of normative cognitive aging that may lead to a worsened ability to problem solve (Harada et al., 2013), thus making older adults more likely to seek or receive advice (Bailey et al., 2021). Older adults have also been shown to express discomfort with their ability to give advice (Strom & Strom, 2017). For instance, of the adults surveyed by Strom and Strom, only 50% felt comfortable giving advice to their grandchildren and 40% felt it was not their place to give advice to their grandchildren or to wait until it was solicited. Nonetheless, advice may be a tool older adults can use to give social support, especially during times in their lives when they may be receiving more support than usual and are unable to reciprocate at the same levels as when they were younger (Krause et al., 1992; Schafer & Upenieks, 2016; Thomas, 2010).

There is a dearth of research focusing on specific advice that older adults give during crises. However, insights from older adults’ advice during times of stability may provide thematic clues. For example, Nimrod and Ben-Shem (2015) conducted a study on advice given to students on how to age successfully. Notably, the older adults in this study based their advice on their own lived experiences. This sample of older adults emphasized developing meaningful relationships, prioritizing health, and developing healthy internal resources like spirituality and optimism. These findings are consistent with characteristics of older adults who tend to be more religious (Bengtson et al., 2015), have higher levels of well-being (Blanchflower & Oswald, 2008), and higher levels of social satisfaction (Charles & Carstensen, 2010) compared to younger adults. Thus, the proscribed advice older adults give is mirrored in their own experiences about aging well, and offers valuable insight to younger generations who may not be functioning as positively.

### The Present Study

Prior research suggests that older adults may be a source of untapped insight and advice. We posited that advice from older individuals could be particularly helpful during crises such as the COVID-19 pandemic due to the wealth of historical and life experiences older generations have, despite ageist portrayals and discounting of older adults during early months of COVID-19 (e.g., Ayalon et al., 2021). The present study investigated the specific advice older adults would give to others regarding the COVID-19 pandemic if given the chance. We sought to understand the nature of advice offered by older adults through multiple time points during a crisis to explore changes in their given advice. Though exploratory in nature, we hypothesized that advice given by older adults would reflect insights gained from their lived experiences. Further, by exploring advice at two time points (one early in the pandemic and one a year later), we hypothesized there would be a shift in advice offered over time in that participants would utilize their experiences during the pandemic to inform their advice after a year of pandemic life. Having gained new life experiences with the COVID-19 pandemic, we hypothesized this would create new and unique advice between time points. Additionally, wisdom is developed through adversity (Igarashi et al., 2018). Thus, we hypothesized advice given a year into the pandemic would shift or differ from advice given early into the pandemic after having more time to reflect on previously proffered advice and enduring adversity (i.e., loss in autonomy, social connections, fear). For example, given older adults experienced increasing social isolation fatigue across the first year of the pandemic (Fuller et al., 2022), they may have shifted from undervaluing social connections in their early advice to overemphasizing the importance of reestablishing social contact in later advice.

## Method

### Study Sample

The community-based, convenience sample for this study consisted of 72 older adults recruited via newspaper, flyers, social media, and word-of-mouth in North Dakota and Minnesota. Participants were recruited for a four-wave longitudinal study that took place between March 2020, when local COVID-19 precautions began (Kaiser Family Foundation, 2020) and April/May 2021, after a year of enduring the pandemic. Because participants were recruited from a geographic region that spanned two states, participants faced distinctions in practices and policies related to COVID-19. Minnesota enacted various statewide public health policies (i.e., mask or vaccination mandates) at various points of 2020–2021, whereas North Dakota prohibited statewide mandates (i.e., required masking or proof of vaccination) related to COVID-19 precautions (Rough & Markowitz, 2022).

Only two time points of data were included in the current study as questions about advice were posed during Waves 2 (June 2020) and 4 (April–May 2021). Wave 2 consisted of 68 participants and Wave 4 consisted of 56 participants. Of the 72 total participants, 52 participated in both waves.

Participants in the entire sample ranged in age from 70 to 97 years old (*M* = 81.6, *SD* = 7.5), were largely female (73.6%), White (97.2%), and heterosexual (95.8%). The majority of participants lived in North Dakota (62.5%), and the remainder lived in Minnesota (37.5%). More than three-fourths had at least some college education. The majority were widowed (51.4%) or married (34.7%) with slightly more than half living alone. The minority lived in rural communities (36.6%), as determined using zip codes (U.S. Census Bureau, 2020) in which cities with a population larger than 50,000 were considered urban. Sample characteristics across waves are shown in Table 1.

**Table 1.** Participant Characteristics

Variables	Wave 2 (*n* = 68)		Wave 4 (*n* = 56)		Total^a^ (*N* = 72)	
	Mean (*SD*)	*N* (%)	Mean (*SD*)	*N* (%)	Mean (*SD*)	*N* (%)
Age in years	81.49 (7.46)		81.50 (7.19)		81.6 (7.5)	
Female		50 (73.5)		39 (69.6)		53 (73.6)
White		67 (98.5)		56 (100)		70 (97.2)
Lives alone		38 (55.9)		30 (53.6)		40 (55.6)
Lives rurally		25 (36.8)		20 (35.7)		26 (36.6)
North Dakota residency		41 (60.3)		35 (62.5)		45 (62.5)
Education (some college or more)		55 (75.0)		41 (73.2)		55 (76.4)
Heterosexual		65 (95.6)		53 (94.6)		69 (95.8)
Marital Status						
Widowed		35 (51.5)		25 (44.6)		37 (51.4)
Married		23 (33.8)		21 (37.5)		25 (34.7)
Divorced		7 (10.3)		7 (12.5)		7 (9.7)
Never married		1 (1.5)		1 (1.8)		1 (1.4)

*Notes*: *SD* = standard deviation.

^a^Of the 72 total participants, 52 participated in both Wave 2 and Wave 4.

### Data Collection

This study was approved by the Institutional Review Board at North Dakota State University. All participants gave verbal consent prior to each interview. A team of seven researchers, five of which were graduate students, conducted qualitative interviews over the phone that generally lasted between 30–90 min. Variance in length of interviews resulted from differences in talkativeness among participants. All interviewers were trained to use the same interview procedures in advance, and each followed a clear protocol with core questions as well as follow-up prompts and guidelines for encouraging in-depth, detailed responses. At each time point, 4–5 interviewers conducted interviews within a 6-week time period, in an attempt to ensure consistency of historical event timing given everchanging pandemic circumstances. Four participants completed mailed paper surveys at Wave 4 due to hearing problems. Phone interviews were recorded and professionally transcribed verbatim. Semistructured phone interviews have been shown to demonstrate high-quality data when face-to-face interviews are not possible (e.g., Ward et al., 2015). As COVID-19 posed significant risk to the study population, data collection was conducted from a distance via telephone interviews.

During Wave 1 interviews, demographic information was collected from participants including race, home size, age, state of residence, education level, gender, marital status, sexual orientation, and whether they lived in a rural or urban environment. During Waves 2 and 4, participants were asked qualitative questions related to advice provision. During Wave 2, participants were asked, “What advice would you give to others during this pandemic?” During Wave 4, participants were asked, “After a year of this pandemic, what advice would you give to others about how to live life after COVID is over?” During both Waves 2 and 4, participants were asked, “What advice would you give to communities and families about the needs of older adults during this pandemic?” This was the only question posed at both time points as we wanted to document how older adults’ needs might have changed over the course of the pandemic.

### Data Analysis

As a first step of analysis, interview transcripts and copies of mailed surveys were holistically coded (Saldaña, 2016) by two researchers trained in qualitative analysis. For the second step of analysis, the lead author (B. Van Vleet) pattern-coded all data to develop a coding scheme. This method was selected as researchers sought to identify main patterns in the transcripts (Saldaña, 2016). An additional coder (B. Hofmann) then independently coded all data using this coding scheme to independently verify accuracy and reliability. She added eight codes to the scheme. Both coders met to review the additional codes and integrated them into the final coding scheme. Additionally, the final set of codes were found to be similar to other work in the field of advice provision (e.g., Lewis & Kim, 2020; Nimrod & Ben-Shem, 2015). This pattern coding process resulted in 10 codes for advice during the pandemic, 13 codes for advice after the pandemic, and 14 codes for advice regarding older adults and society. To further organize and collapse this comprehensive set of codes, we used Saldaña’s (2016) “trinity” process to merge codes together into three major codes per question. Examples of the coding scheme and trinity process can be found in Table 2.

**Table 2.** Examples of Qualitative Codes for Advice Participants

Theme	Examples of identified codes	Trinity
Theme 1: advice during COVID	Accept the situation Stay optimistic This too shall pass	Develop positive life perspectives
	Connect with others	Connect with others
	Maintain balance between mental and physical health Follow rules Respect others’ boundaries	Foster mental and physical well-being
Theme 2: advice after COVID	Be a better person Forgive Help others	Emerge as better people
	Use caution COVID is ongoing Follow directions of experts	Remain vigilant
	Don’t take life for granted Appreciate Connect or reconnect	Return to normal
Theme 3: older adults and society	Maintain balance	Maintain balance
	Check in/on them Connect with them Continue safety precautions	Take intentional actions
	Pay attention to them Be selfless Respect them	Adopt selfless attitudes

*Note*: COVID = coronavirus disease.

To analyze change over time, the findings from each time point were analyzed separately and the resulting codes were compared across time points to identify differences. No aggregate differences were found. We also investigated individual differences using transcripts from each time point compared side by side. Individuals who offered different advice at each time point were identified. Finally, the codes from those individuals’ transcripts were compared against each other to determine if any clear patterns emerged.

## Results

### Advice for During the Pandemic

Analyses revealed three major themes in older adults’ advice for others during the early months of the COVID-19 pandemic (Time 2): foster mental and physical well-being, promote positive life perspectives, and prioritize connections and maintain social relationships. Older adults advised individuals to *foster both mental and physical well-being*. Participants emphasized that staying healthy despite the pandemic was important. For example, a 76-year-old female participant said, “Practice safety, personal safety. I mean any states that have opened up, the ill people count is going up tremendously and will continue to. You know, the thing to do is obey what the scientists are telling us.” Thus, some participants indicated that some personal safety measures were still necessary to promote physical wellness, despite governments relaxing certain societal prevention measures such as reopening after periods of lockdown. However, participants also cautioned that physical safety should not come at the cost of mental well-being. As one 77-year-old female participant stated, “I guess it doesn’t pay to worry. Do the best you can. You know, try and follow some of the safe rules, but you can’t let it take over your life. You know you got to live too.” This participant noted that physical well-being was important, but that obsessing over physical well-being to the point of sacrificing mental well-being (i.e., “worrying”) was not healthy either. This balance between physical and mental health was also highlighted by a different 77-year-old female: “Keep yourself safe, but not be paralyzed with fear and stay inside and not do anything because you can’t survive that way either.” Again, participants stressed that both physical and mental well-being were important, and advised against emphasizing one over the other.

The second identified theme of *promoting positive life perspectives* encompassed advice related to seeking a positive outlook. For example, many participants advised others to take each day one at a time or to rely on faith. One 96-year-old female participant advised, “One day at a time and trust in the Lord, He’ll take care of us.” This participant emphasized utilizing faith and staying present as strategies people could employ to cope through the pandemic. Others, like this 71-year-old male, emphasized optimism saying, “Look for the bright side. Look past today, look for tomorrow, because it will pass. The flu epidemic in 1918 passed too and that killed a much higher percentage of our population than this one ever will.” By recalling past events, others could have an optimistic and grounded view of their current situation despite adversity. A 75-year-old woman advised acceptance of the situation by stating, “I just think that they must accept it the way it is and try to live and just try to do your best.” Again, this participant endorsed harboring a positive life perspective by advising others to accept the situation, rather than mourn what was missing.

Finally, older adults recommended that people *prioritize connection with others and maintain social relationships*. One 75-year-old male said, “I would say don’t wait for other people to reach out to you. Be proactive to maintain social interactions.” Others, like this 86-year-old female, noted that outreach can be beneficial for the recipient as well: “Stay in touch with people. I mean other people, if you know somebody that is alone. Even if they have relatives, it doesn’t hurt to get a call from someone by surprise which would be nice. Think of others, maybe.” In both cases, the participants indicated that social relationships during the pandemic required effort. The second participant emphasized that putting forth an effort to connect was fruitful as it was kind to others. A different 75-year-old man related his advice back to his own coping: “Try to reach out to other people by phone or social media or whatever it is. Stay connected. And I have done that, you know, so it’s not that I’m completely isolated. I do at least interact on the phone and sometimes in person with other people.” Thus, this participant not only advised social contact, but also practiced it in his daily life.

### Advice for After the Pandemic

Analyses of responses after a year of the pandemic (Time 4) revealed three themes of older adults’ attitudes regarding postpandemic life. First, some older adults took issue with the idea that the pandemic will pass. They urged others to *remain vigilant*. In some cases, participants worried that a lack of vigilance would lead to the virus becoming an ongoing reality. In short, they recommended that communities remain vigilant as they adjust to the new normal. One 78-year-old male expressed this view by saying, “I’m not sure COVID is going to be a thing of the past. I don’t think we will go back to life the way we had it. I don’t think we can expect to.” Others gave the impression that COVID-19 would end but another pandemic would crop up in its place. For example, an 88-year-old woman said, “Once we think it’s over, there’s something else that will come up. We just have to be ready for the next one.” Another perspective was that a lack of vigilance would lead to the emergence of another pandemic or the reinstatement of pandemic-related precautions. One 71-year-old woman advised, “I think, listen to the guidelines, follow the rules, so this doesn’t have to happen again.”

Secondly, participants advised *returning to normal*. Some participants felt that the pandemic was over and a return to prepandemic activities should be a priority. For context, this wave of data collection occurred in May 2021, after the adult vaccine rollout. One 80-year-old woman emphasized reigniting social contact saying, “To renew their contact with people as soon as they can. It’s been a long separation.” Others noted that a return to normal should be gradual but steady. One 87-year-old woman said, “So just be open to dealing with how things come about. And I think it’s going to be gradual, and just keep on coping with things.” Others stressed a positive attitude that individuals should harbor in returning to “normal” life. A 92-year-old man advised, “I’d say enjoy it, I guess. Enjoy the freedom to be able to come and go and do things.” Thus, this second type of advice was future-focused with recommendation to find ways to return to normal activities.

Finally, participants suggested that the public should not simply return to normal but rather *emerge from the pandemic as better people*. Some older adults suggested that the pandemic should change our way of living for the better. Once the pandemic is over, people should not settle for returning to normal but contemplate what kind of world they want to return to. For example, an 82-year-old woman remarked, “I think we just have to be better people coming out of this than we went in. I mean, things like hoarding and quarreling about masking, and getting ugly with other people who think differently. The world is too fragile to not pay attention and help each other out.” Similarly, a 92-year-old woman advised, “Be cautious of what you do and what’s going on in the country. I think try to learn instead of have greed. I think our greed and hatred is growing in the world too much.” Other older adults stressed the importance of learning from the pandemic. For example, learning to not take life for granted. One 80-year-old woman said, “To be grateful for whatever time you have. Seize the moment. Don’t be looking into the future but enjoy life for what it is right now.”

### Advice About Older Adults’ Needs

Our analysis revealed three overarching themes related to advice about older adults’ needs: adopt selfless attitudes, take intentional actions, and maintain balance. First, participants advised that people in society need to *adopt selfless attitudes* by paying attention to older adults. For example, one 92-year-old woman at Time 4 highlighted the differences between her age group and the younger generation by remarking, “If something goes on, it’ll affect the older adults more than anything else. So, if you have adult families, kind of look out for them and be aware that their needs might be more than yours.” Other participants echoed this advice and pointed to lifestyle factors of younger adults that they should account for, such as being busy. A 75-year-old woman at Time 2 recommended, “Try not to be so busy that they can’t look after their people.” At Time 4, a 78-year-old man was even more direct. He admitted he was frustrated with his kids not calling him and expressing interest in his life before saying, “(My kids are) so enmeshed in their own lives, they really don’t look out beyond that. That’s a kind of loneliness, too, when someone doesn’t show interest in who you are and what you’re doing. So that would be advice to the younger people: to listen, to ask questions that elicit a response, and then listen.” It is not enough to have selfless attitudes; those attitudes need to be acted upon.

Participants recommended *taking intentional actions* such as taking the initiative to check on older neighbors and relatives. In our sample, many felt the onus of connection was often on older adults. For example, at Time 2 one 78-year-old man said, “Older people get tired of doing all the reaching out.” Aside from checking on them, older adults also pointed to instrumental help. At Time 2, a 92-year-old woman instructed, “Do whatever you can to help them. This is something I am guilty of. I know I should be sending cards. Once in a while I’ll send cards to give to people in the nursing homes because I really do feel for them. But do something for the old people to help them out.” Thus, some participants infused their advice with reflections from their own life and then evaluated how well they were following their advice. A specific way communities could aid older adults was through grocery shopping. As a 77-year-old woman said at Time 2, “Make sure that they have your phone number in case they need help. Everybody should just become a little more aware of each other or ways that you can help out. (Ask older adults,) ‘Could you use a loaf of bread or some milk?’”

However, participants stressed they were still adults, capable of taking care of themselves and maintaining their autonomy. Thus, *maintaining balance* was the final theme identified in participant responses. An 82-year-old woman said at Time 2, “I almost think they’ve overdone it but they’re doing it out of concern for me.” This participant acknowledged that the people in her life were acting on their selfless attitudes through intentional action, but that the actions were unnecessarily frequent. At Time 2, a 78-year-old man echoed this experience more bluntly saying, “Just check on them. But not too often. Don’t be a pest.” Additionally, many young-old adults did not feel their age made them needier than others. At Time 4, a 77-year-old woman said, “I think the older adults need exactly what any human being needs,” and also at Time 4, a 75-year-old woman quipped, “My kids check on me all the time and I don’t consider myself old.” Thus, many participants noted that communities needed to be proactive in helping to respond to older adults’ needs, but they had diverse opinions on what those needs were. Some prioritized autonomy while others longed for greater community engagement.

### Longitudinal Findings

Despite the longitudinal design, there was not a noticeable difference in advice about older adults’ needs between the two time points. For example, a 77-year-old female gave roughly the same advice at both time points. At Time 2 she said, “I guess kind of check on them and I’m not good about doing that so much myself personally,” and at Time 4 she said, “Well, I suppose check on them a little bit. Make sure that they’re safe. Then again, I don’t know how good I am about doing that.” As another example, an 87-year-old female said at Time 2, “Just stay in touch, even if they don’t. (Ask them) ‘Do you want to go to the grocery store,’ or, ‘Is there anything I can get you?’” and at Time 4 she advised, “I would say contact. You don’t have to go every day, even every week. But if you do know somebody, (ask) ‘Look, I’m free today and I’m going to the grocery store. If there’s something you need, would you like to ride along with me?’” Most participants indicated the exact same thematic advice at both time points.

A small minority, 12 participants, offered thematically different advice between time points. For example, a 77-year-old woman advised *adopting selfless attitudes* at Time 2 saying, “Well I think it is very important for communities and families to be very aware that older adults, especially older adults with underlying conditions, are much more likely to die from this,” but at Time 4 suggested society should *maintain balance*, “I think the older adults need exactly what any human being needs.” A 76-year-old woman also changed her advice saying at Time 2, “Just if there’s someone that needs some help, please do so,” which was coded as *take intentional actions* but at Time 4 advised, “Enjoy them and appreciate them while they’re here” which was coded as *adopt selfless attitudes.* As only a small minority of participants switched their advice between time points, no clear thematic patterns emerged.

An alternative interpretation of these findings is that these 12 participants may have offered the same advice at a later time point if the question had been more pointed. However, because the question was broad, these participants provided different advice. Regardless of how this is interpreted, the majority of participants did not differ or alternate.

## Discussion

The purpose of this study was to examine what specific advice older adults would offer their peers, families, and communities about how to cope during and after the pandemic as well as how to address the needs of older adults in times of crisis. Throughout the pandemic, *connection*, *thinking of others*, and *remaining cautious* were salient themes in older adults’ advice. The significance and implications of each of these themes is discussed next.

COVID-19 warranted precautions that posed social isolation risks for older adults (Webb, 2021). Isolation is associated with negative mental and physical health outcomes in later life (Novotney, 2019; Shankar et al., 2011). Conversely, social connection during the pandemic has been associated with lower stress and pandemic-related worries for older adults (Nitschke et al., 2021; Ungar et al., 2022). Thus, isolation as a risk, and connection as a resilience factor have been established in recent COVID-19 literature. The current findings confirmed this prior research with pervasive themes of connection. Older adults advised connection as a key strategy to cope through the pandemic. Similarly, older adults prioritized reestablishing contact with others once the pandemic passes. Further, one of the intentional actions that older adults advised their communities to take was connecting with and checking on older adults. Older adults’ repeated emphasis on connection throughout the pandemic suggests they understood the benefit of social connection during and following the pandemic—for themselves and for their community. This emphasis may have resulted from increased isolation which highlighted the need for and benefit of social connections (Fuller et al., 2022). Further, it is interesting to note that due to the unique geographic location of this sample, they may be well versed in navigating patterns of isolation and reconnection due to harsh winters which make local travel difficult (Bjelde & Sanders, 2009; Hjorthol, 2013), especially among those in sparse or remote communities (Scott, 2010). Middle America is also generally friendly and sociable (Rentfrow et al., 2013); thus, it is not surprising that these participants advised social connection as a prime coping strategy, whereas other geographic regions may prioritize advising different coping methods depending on local culture and climate. Thus, our findings related to both themes of isolation and connectedness may be particularly unique in this sample.

Altruism has been shown to act as a weak protective factor against decline in older adulthood (Greenfield, 2009) and is associated with lower morbidity (Brown, et al., 2005). In this study, older adults overwhelmingly encouraged people to be aware of others’ needs during the pandemic. As previously mentioned, older adults stressed communication with others as a way to cope through the pandemic. Often, this theme of communication was merged with altruism. For example, participants such as the 86-year-old female highlighted earlier, emphasized connection generally, but specifically for those who were alone and thus, more in need of connection. Additionally, the first theme in advice about older adults’ needs, *adopt selfless attitudes,* connects to altruism. To adequately care for older adults during the pandemic, one needs to be altruistic about their needs. The theme of *emerging as better people* often involved thinking of others and helping to build a less selfish world. These findings suggest that altruism is a salient theme of older adults’ worldviews in times of crisis. Future research should evaluate whether giving advice has similar positive health benefits as other forms of altruism and generativity, particularly outside of a research setting.

Previous scholars highlighted concerns about consistent messaging about older adults’ vulnerability during the pandemic (e.g., Ayalon et al., 2021; Webb, 2021). When asked for advice after the pandemic passed, participants stressed to *remain vigilant*. The emphasis on vigilance from this study’s participants in spring 2021 came after vaccine rollout and an ease in restrictions such as stay-at-home orders and other advised precautions. This timing suggests that older adults may be more cautious about getting sick from the COVID-19 virus compared to government leaders and others in their community who emphasized reducing restrictions. Alternatively, older adults may be more cognizant of the general negative health effects of aging, after COVID-19 messaging highlighted their vulnerability (Seifert, 2021).

Older adults are effective at coping (Neubauer et al., 2019) in large part due to their life experience (Aldwin & Igarashi, 2016; Neupert & Bellingtier, 2019). In this study, older adults’ advice seemed to draw from their life experiences and older adults integrated their own coping strategies to their advice. Participants suggested people should rely on faith, accept the circumstances and live in the moment, and connect with others—strategies they employed in their own lives during the pandemic (see Fuller & Huseth-Zosel, 2021). This study further contributes to the evidence that older adults build their coping strategies through a lifetime of experiences and provides evidence that coping skills could be shared intergenerationally through advice-giving.

The longitudinal approach of this study offers insight into the wisdom and advice offered by older adults over time. Notably, older adults’ advice to others about their own needs did not noticeably change throughout the pandemic. Despite ageist messaging that all older adults are equally vulnerable to COVID-19 and its effects (e.g., Ayalon et al., 2021; Graf & Knepple Carney, 2021; Webb, 2021) which can lead to increased risk aversion, learned helplessness (Coudin & Alexopoulos, 2010), and compassionate ageism (Vervaecke & Meisner, 2020), community-dwelling older adults advised a balanced approach from community and family members responding to their needs and the needs of their peers. Older adults who are more vulnerable to the effects of COVID-19, such as those living in care facilities, may proffer different advice. This lends support to an advised approach to empower older adults to vocalize their care needs as an individual rather than as an age-group monolith to prevent learned helplessness (Faulkner, 2001).

Participants in this study indicated that older adults need connection but are still capable of caring for themselves and maintaining independence. These findings contrast Newton and colleagues (2021) who found that older adults desired more assistance in completing basic tasks during the pandemic. These themes were salient at both time points, suggesting they were cognizant of the need for balance regardless of the prevalence of COVID-19 precautions. Additionally, these findings suggest that many older adults are cognizant of their needs and can vocalize them. Overall, the findings of this study indicate that older adults have a nuanced understanding of their vulnerability and resilience. They are aware they are at-risk inasmuch as they are aware they are capable of enduring. Older adults are not helpless.

The similarity between advice toward the beginning of the pandemic and 1 year into the pandemic was not consistent with the shift we hypothesized that older adults would integrate their lived experience throughout the pandemic to reinform their advice later in the pandemic. Instead, advice during the pandemic consistently focused on strategies to cope with and survive the crisis. However, when asked for advice on how others should live after the pandemic passed, some participants still maintained the importance of surviving through subsequent pandemics or acknowledged they did not think this pandemic was close to being over. These themes were present despite the decidedly future-tense framing of the question. These findings suggest that older adults may be more focused on the present than the future amid a perceived crisis and are not able to conceptualize a crisis-free future. Alternatively, it is possible that the alarming and dominant news coverage of the pandemic (Ng et al., 2021) and increased consumption of news media being associated with more psychological distress (Stainback et al., 2020) contributed to the trend of persistent future-oriented pessimism. However, some research has suggested that increased news consumption during the pandemic improved mental health (Villasanta et al., 2022). Thus, future research should seek to better understand these findings.

Although analyzing these findings through the lens of wisdom is beyond the scope of this paper, future research should also evaluate the amount of wisdom present or missing from older adults’ advice. As not all older adults are wise (Ardelt, 2011), investigating what advice is considered wise and unwise may be helpful to cultivate and share wisdom intergenerationally.

### Limitations

This study offers rich longitudinal qualitative data on the unique, underinvestigated topic of advice-giving among older adults; however, there are several important limitations. This study represents community-dwelling older adults living in both rural and urban areas of North Dakota and Minnesota; thus, these findings are not generalizable to a national population. Further, this research represented participants who were mostly middle-to-upper class White women who were well-educated. Therefore, the advice offered by lower income and minority populations may have greatly differed from the participants in this study. Additionally, the advice in this paper was directly solicited from older adults in the context of a research interview. The presence and substance of advice given spontaneously to community members, family members, or peers may differ or not appear at all. Also, we utilized multiple interviewers over the 1-year study which may have affected the reliability of our interviews. Finally, historical crises that differ significantly from the COVID-19 pandemic may yield different types of advice. Further research is needed to better understand the content of older adults’ advice and distinctions between advice offered generally during historical crises and advice specific to the COVID-19 pandemic context.

### Directions for Future Research and Applied Work

The themes from this exploratory study should be further investigated by researchers to determine what themes of older adults’ advice-giving are specific to COVID-19 and what is universal. This study suggests that despite changes in the context of COVID-19 over the course of a year, the advice older adults offered did not shift. As older adults utilize wisdom to help younger generations solve problems (Parisi et al., 2009; Tabuchi & Miura, 2015), understanding the themes of advice directed toward specific problems, and what is given generally, may provide insight on the ways older adults’ insight and wisdom could be applied to a variety of problems if solicited. There is potential for such applied advice to increase older adults’ feelings of generativity and well-being (Tabuchi & Miura, 2015; Thomas, 2010). Additionally, advice-giving has been linked to improved feelings of control and greater life meaning (Schafer & Upenieks, 2016) in older adulthood (Krause et al., 1992).

Future research should also evaluate how willing the public is to listen to older adults. There is some evidence that not feeling respected by younger generations may lead to a decrease in engaging in generative behaviors (Cheng, 2009). As a goal of adulthood is achieving generativity (Tabuchi & Miura, 2015) and older adults have been shown to benefit from advice-giving (Krause et al., 1992; Schafer & Upenieks, 2016; Thomas, 2010), understanding how older adults can effectively disseminate advice to others in the general public may provide more meaningful opportunities for older adults to share their wisdom with their communities. Additionally, future research should evaluate whether coping skills can be shared, taught, and learned intergenerationally through advice-giving. Finally, future research should seek to evaluate how older adults perceive the quality of their own advice. Understanding this may help develop interventions to understand how ageism, both internalized and externalized, affects advice-giving.

## Conclusion

This study suggests that older adults are a source of insight during historical crises such as the COVID-19 pandemic. Older adults are equipped with life experience that enables them to vocalize their own needs, provide advice related to coping strategies for present troubles, and guide others into the future.
